# ELDER FINANCIAL ABUSE IN FAMILIES: EXPANDING THEORY AND RESEARCH

**DOI:** 10.1093/geroni/igz038.1406

**Published:** 2019-11-08

**Authors:** Cory Bolkan, Marlene Stum, Pamela B Teaster

**Affiliations:** 1 Washington State University, Vancouver, Washington, United States; 2 University of Minnesota, St. Paul, Minnesota, United States; 3 Virginia Tech, Blacksburg, Virginia, United States

## Abstract

Elder family financial exploitation (EFFE) is widespread and increasing. The effect is devastating, causing significant financial losses, reducing health and well-being of elders, and disrupting family systems. Research reveals that most (90%) perpetrators are family members or trusted others and researchers typically focus on identification of the problem, rather than understanding how and why exploitation occurs within the family unit. Furthermore, limited consensus exists regarding a theoretical understanding of the complexities of EFFE. Theory-driven, empirical explanations of how and why EFFE transpires are urgently needed to enhance and deepen intervention and prevention efforts. In this symposium, we extend both theory and research by using a common theoretical lens to present research findings from three distinct EFFE studies. The first paper reviews the current literature on EFFE and theory and introduces Bronfennbrenner’s bioecological Process-Person-Context-Time (PPCT) model as an under-utilized, but useful framework for understanding EFFE. The second paper reports on findings from in-depth interviews with non-perpetrator family members who experienced EFFE and will highlight complex intergenerational family systems processes in PPCT. The third paper highlights findings from a national study of substantiated and investigated cases of EFFE in which family member perpetrators were designated as surrogate decision makers. The fourth paper shares results from a qualitative study of family member POAs and how components of the PPCT model can be interpreted for use by helping professionals assisting families. We will also focus on the opportunities and challenges of developing theoretically sound EFFE research and the implications for improving practice and policy.

